# Long-Term Exposure to Oroxylin A Inhibits Metastasis by Suppressing CCL2 in Oral Squamous Cell Carcinoma Cells

**DOI:** 10.3390/cancers11030353

**Published:** 2019-03-12

**Authors:** Wei-Ting Ku, Jiun-Jia Tung, Tony Jer-Fu Lee, Kuo-Chu Lai

**Affiliations:** 1 Master Program of Pharmacology and Toxicology, Department of Medicine, School of Medicine, Tzu Chi University, Hualien 97004, Taiwan; 106721104@gms.tcu.edu.tw (W.-T.K.); 103721102@gms.tcu.edu.tw (J.-J.T.); 2 Department of Pharmacy, Yuli Hospital, Ministry of Health and Welfare, Hualien 98147, Taiwan; 3 Department of Medical Research, Buddhist Tzu Chi General Hospital, Hualien 97004, Taiwan; tlee@mail.tcu.edu.tw; 4 Cardiovascular and Metabolomics Research Center, Buddhist Tzu Chi General Hospital, Hualien 97004, Taiwan; 5 Department of Pharmacology, Tzu Chi University, Hualien 97004, Taiwan

**Keywords:** oroxylin A, oral squamous cell carcinoma, CCL2, metastasis

## Abstract

Oroxylin A (Oro-A), the main bioactive flavonoid extracted from *Scutellaria radix*, has been reported to inhibit migration in various human cancer cell models. In this study, we further explored the anti-migration effects of Oro-A on oral squamous cell carcinoma (OSCC) cells and investigated the underlying mechanisms. A 24-h (short-term) exposure of OSCC cells to non-cytotoxic concentrations (5–20 μM) of Oro-A significantly suppressed cell migration according to a wound-healing assay. Furthermore, a 30-day exposure (long-term) to Oro-A (20 μM), which did not exhibit a cytotoxic effect on OSCC cells, significantly suppressed cell migration more than short-term Oro-A exposure. To uncover the molecular mechanisms underlying the inhibitory effect of long-term Oro-A exposure on OSCC migration, a cDNA microarray and the Ingenuity software were used. Overall, 112 upregulated and 356 downregulated genes were identified in long-term Oro-A-exposed cells compared with untreated OSCC cells. Among them, 75 genes were reported to be associated with cancer cell migration. Consistent with the cDNA microarray results, we found that the expression levels of several cell migration-related genes, such as LCN2, ID-1, MDK, S100A9 and CCL2, were significantly decreased in long-term Oro-A-exposed OSCC cells using a quantitative real-time polymerase chain reaction (Q-PCR) assay. The Western blotting and enzyme-linked immunosorbent assay (ELISA) results also demonstrated that CCL2 expression at the mRNA and protein levels was significantly decreased in long-term Oro-A-exposed OSCC cells compared with untreated OSCC cells. Moreover, the expression levels of downstream CCL2 targets, including p-ERK1/2, NFκB, MMP2, and MMP9, were also decreased in long-term Oro-A-exposed OSCC cells. Further, Oro-A treatment suppressed in vivo metastasis. These results suggest that long-term Oro-A treatment inhibits metastasis via CCL2 signaling in OSCC cells.

## 1. Introduction

Oral cancer is the most frequent type of cancer of the head and neck area, with oral squamous cell carcinoma (OSCC) being the most common single entity [1,2]. Advances in surgical technique and preoperative management have improved the survival rate to some extent; however, the overall 5-year survival rate for OSCC patients remains at 50%, without significant improvement over the past three decades [3]. The major causes of OSCC-related death are cervical node and distant metastasis [4]. Metastasis of OSCC is a complex process involving detachment of cells from tumor tissue, regulation of cell motility, invasion, and proliferation and evasion through the lymphatic system or blood vessels [1]. Thus far, limited therapeutic choices are available for OSCC patients with metastatic disease.

The inflammation response occurs via microbial infection, exposure to allergens and toxic chemicals, obesity, and autoimmune disease [5,6]. Cancer-related inflammation has been considered the seventh hallmark of cancer [7]. The key characteristics of cancer-related inflammation include infiltration of white blood cells; prominence of tumor-associated macrophages; the presence of polypeptide messengers of inflammatory cytokines, such as tumor necrosis factor (TNF), interleukin (IL)-1, and IL-6; chemokines, such as CCL2; and the occurrence of tissue remodeling and angiogenesis [7]. Emerging evidence has shown that cancer-related inflammation contributes to the proliferation and survival of malignant cells, angiogenesis, metastasis, disruption of adaptive immune responses, and depressed responses to hormones and chemotherapeutic agents [6,7,8]. Numerous inflammation-mediated molecular pathways have been explored and studied as important factors in OSCC carcinogenesis, such as cyclooxygenase (COX)-2, epidermal growth factor receptor (EGFR), p38a MAP kinase, TNF, NF-kB, STAT, RhoC, and PPARy pathways. [9]. Similar to other cancer types, cancer-related inflammation represents a therapeutic target in OSCC.

Flavonoids, polyphenolic compounds derived from plants, have a broad spectrum of biological activities, including antioxidant, anti-inflammation, and anticancer activities [10,11]. Because of their multiple biological activities and their safe toxicological profile, flavonoids have been widely studied in the last decade as potential leads for anticancer therapy [10]. Oroxylin A (Oro-A, 5,7-dihydroxy-6-methoxyflavone), is the major bioflavonoid of *Scutellaria radix* [12]. As a natural product, Oro-A has several advantages, including high selectivity between normal cells and cancer cells and low toxicity [13]. It has been shown that Oro-A exerts a broad spectrum of pharmacological functions, including anti-inflammation, neuroprotective, anti-virus and anti-coagulation activities [14,15,16,17,18]. In addition, Oro-A shows a reliable anticancer effect on several cell lines, such as those derived from lung cancer [19], breast cancer [20], gastric cancer [21], hepatoma [22], colon cancer [23], cervical cancer [24], and leukemia [25]. The antitumor activities of Oro-A have been related to effects on apoptosis [26], differentiation [22], cell cycle arrest [21], and migration/invasion [26,27,28]. However, no study has attempted to understand the anti-tumor effects of Oro-A in OSCC.

In the present study, we explored the anti-migration activity of Oro-A at non-toxic concentrations. We further analyzed expression profile changes to investigate the mechanisms underlying the anti-migration effect of long-term Oro-A exposure and demonstrated the involvement of CCL2 in the anti-migration activity of long-term Oro-A exposure in OSCC. Finally, we demonstrated the effect of Oro-A on OSCC metastasis in vivo.

## 2. Results

### 2.1. Long-Term Exposure to Oro-A Significantly Inhibited Migration of OSCC Cells with Non-Cytotoxic Effects

**Figure 1:**
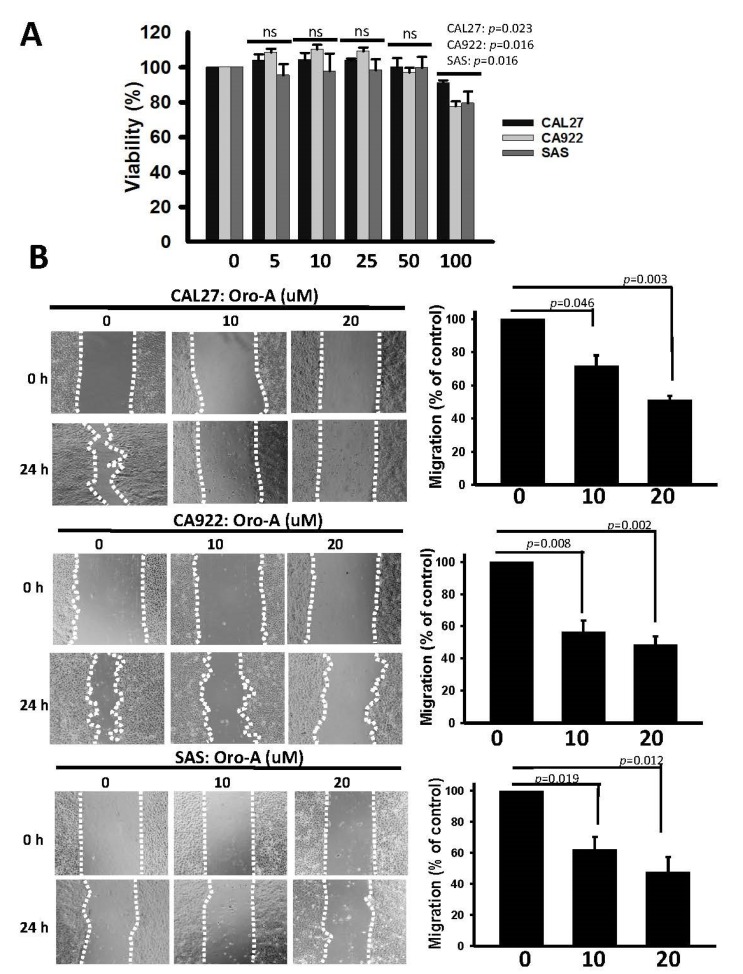
Effect of Oro-A exposure on the migration activity of oral squamous cell carcinoma (OSCC) cells. (**A**) CAL27, CA922, and SAS cells were treated with the vehicle control (dimethyl sulfoxide, DMSO) or Oro-A (0–100 μM) for 72 h, and relative survival was assessed with a sulforhodamine B (SRB) assay. (**B**) OSCC cells were treated with vehicle (DMSO) or Oro-A (10 and 20 μM) for 24 h, and the migration activity of cells was determined with a wound healing assay. All experiments were performed at least three times. P values were determined using Student’s t test. Ns: not significant.

The cytotoxic effect of Oro-A on OSCC cells was determined using a sulforhodamine B (SRB) assay (Figure 1A). Oro-A did not effectively inhibit the cell viability of OSCC cell lines, including CAL27, CA922 and SAS, until a concentration of 100 μM. Moreover, we examined the effect of Oro-A on cell migration under non-toxic concentrations using a wound-healing assay. As shown in Figure 1B, Oro-A dose-dependently significant reduced wound healing migration ability in OSCC cells, indicating that short-term Oro-A exposure did not affect cytotoxicity but could inhibit OSCC migration ability.

**Figure 2:**
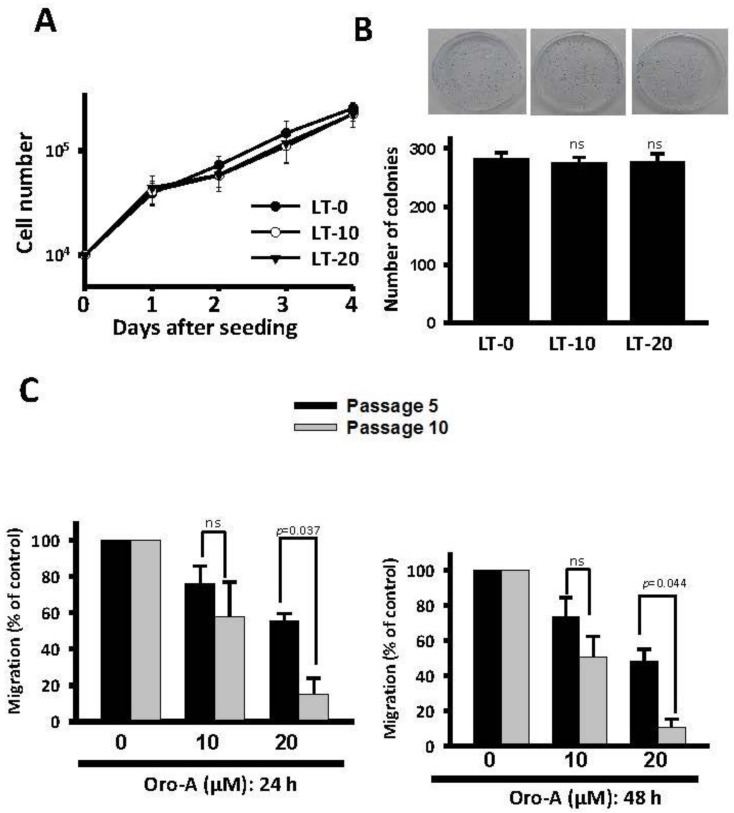
Long-term effect of Oro-A on the migration activity of OSCC cells. CAL27 cells were treated with vehicle (DMSO) or long-term exposure to Oro-A (10 and 20 μM) for 10 passages. Long-term Oro-A-exposed OSCC cells were designated LT-10 and -20 cells. The growth rates of LT-10 and -20 cells were analyzed with (**A**) trpan blue dye exclusion and (**B**) colony formation assays. (**C**) The migration activity of long-term Oro-A-exposed cells (5 and 10 passages) was determined with wound healing assays. All experiments were performed at least three times. P values were determined using Student’s t test. Ns: not significant.

To investigate the effect of long-term Oro-A exposure on growth rate and migration abilities, we exposed OSCC cells to non-toxic Oro-A doses (0, 10, and 20 μM) for 10 successive passages (30 days). These long-term Oro-A-exposed OSCC cells were designated LT-0, -10, and -20 cells, respectively. As shown in Figure 2A,B, no marked changes in proliferative rate were observed after long-term Oro-A treatment based on trypan blue exclusion and colony formation assays. We further evaluated the migration ability of cells subjected to long-term Oro-A exposure using a wound-healing assay. As shown in Figure 2C, the inhibitory effect of Oro-A exposure on cell migration after 5 passages exposed to non-toxic Oro-A doses (0, 10, and 20 μM) was similar to that of a 24-h treatment. At 24 h after wound generated, exposure to 20 μM Oro-A for 10 passages significantly inhibited migration more than exposure for 5 passages. The same result was obtained at 48 h after the wound was generated, further confirming that the inhibitory effect of long-term Oro-A exposure on cell migration. These results demonstrate that long-term exposure to Oro-A did not affect growth rate but could inhibit migration ability better than short-term exposure.

### 2.2. Migration-Related Genes Were Validated in Long-Term Oro-A-Exposed OSCC Cells

**Figure 3:**
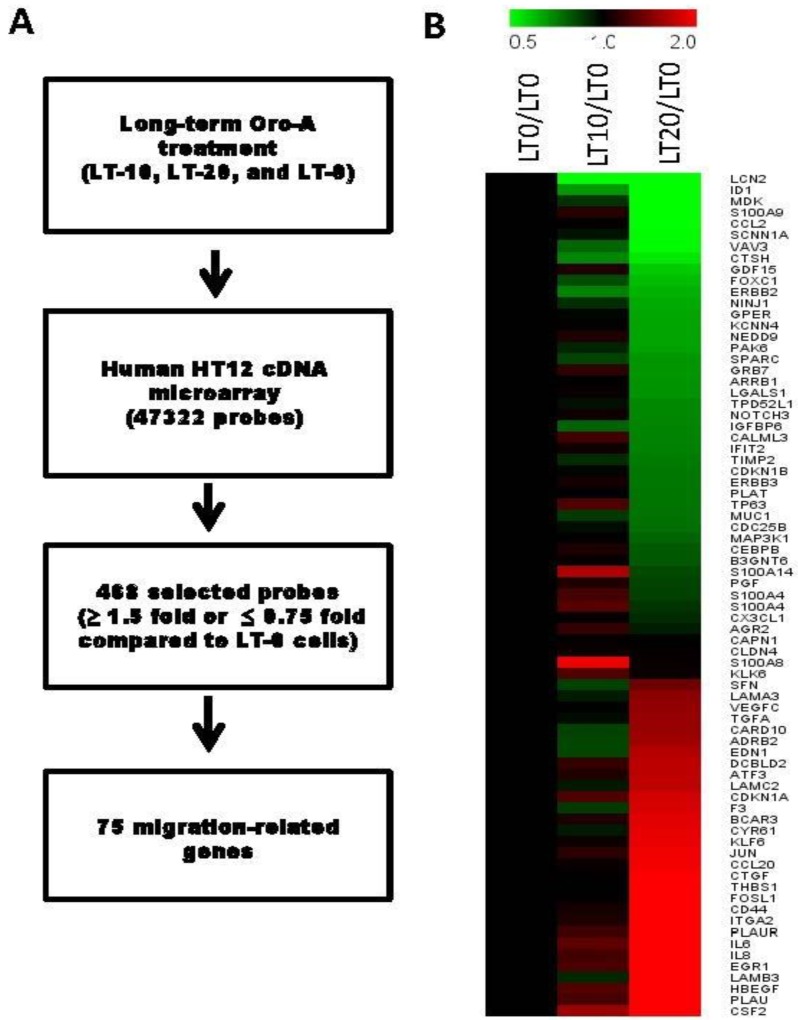
cDNA microarray analysis of gene expression profiles in long-term Oro-A exposed OSCC cells.(**A**) Schema for the identification of the migration-related genes affected by long-term exposure to Oro-A. (**B**) Heatmaps showing 75 selected migration-related genes from LT-20 cells. Red represents increased expression; green represents decreased expression.

**Figure 4:**
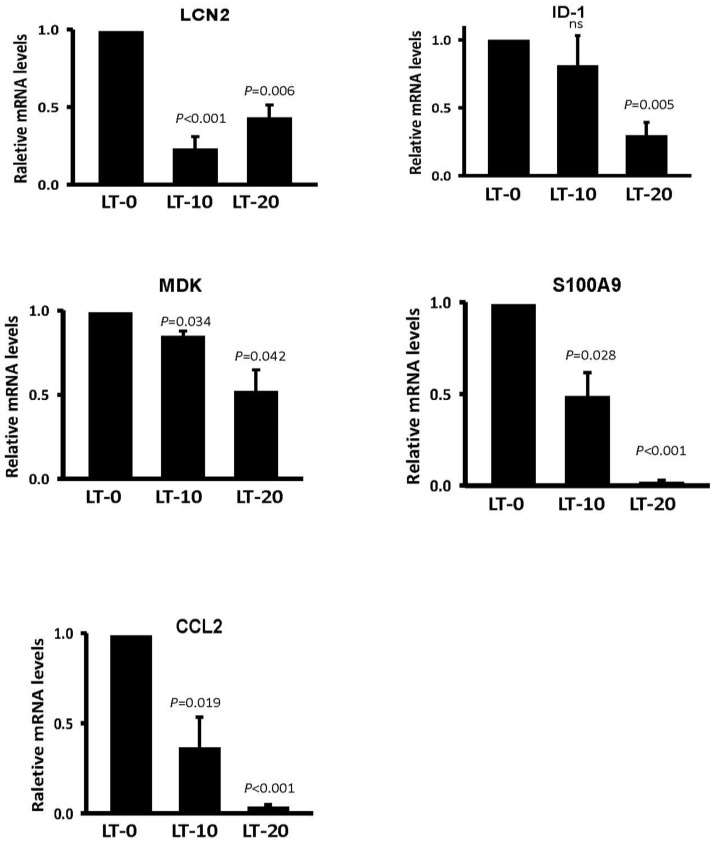
Five migration-related genes were validated in long-term Oro-A-exposed OSCC cells. The mRNA levels of LCN2, ID-1, MDK, S100A9, and CCL2 were determined by Q-PCR. Beta-2-microglobulin (β2M) was used as the internal control. All experiments were performed at least three times. P values were determined using Student’s t test. Ns: not significant.

To determine differences in the expression of migration-related genes after long-term Oro-A exposure in OSCC cells, HumanHT-12 v4 Expression BeadChip was utilized. LT-0 cells served as the control for determination of expression profile changes. Genes with expression ratios that were at least 1.5-fold higher or 0.75-fold lower in LT-20 cells were selected. In total, 112 upregulated probes and 356 downregulated probes were selected (Figure 3A). According to Ingenuity Pathway Analysis (IPA) software, 75 genes have been reported to be related to cancer cell migration (Figure 3B). The top 10 downregulated and upregulated migration-related candidate genes are shown in Table 1 and Table 2, respectively. According to the IPA prediction, several downregulated genes, such as *LCN2, ID1, MDK, S100A9*, and *CCL2*, were predicted to potentially decrease migration in long-term Oro-A exposure OSCC cells. However, most of the upregulated genes were predicted to enhance migration after long-term Oro-A exposure. Thus, we focused on the top 5 downregulated migration-related genes, namely, *LCN2, ID1, MDK, S100A9*, and *CCL2*, which may be related to the migration inhibition induced by long-term Oro-A exposure. The mRNA levels of these five migration-related genes were further confirmed by quantitative real-time polymerase chain reaction (Q-PCR). As shown in Figure 4, we found that long-term Oro-A exposure significantly reduced *LCN2, MDK, S100A9*, and *CCL2* mRNA expression in LT-10 and -20 cells compared with LT-0 cells. However, a 24-h treatment with Oro-A (20 μM) had no significant effect on the expression of *LCN2, ID1, MDK, S100A9*, and *CCL2* (Figure S1), showing that the mechanism underlying the inhibitory effect of long-term Oro-A exposure on migration may be related to *LCN2, ID1, MDK, S100A9*, and *CCL2* expression.

### 2.3. Long-Term Oro-A Exposure Downregulates *CCL2* Signaling

**Figure 5:**
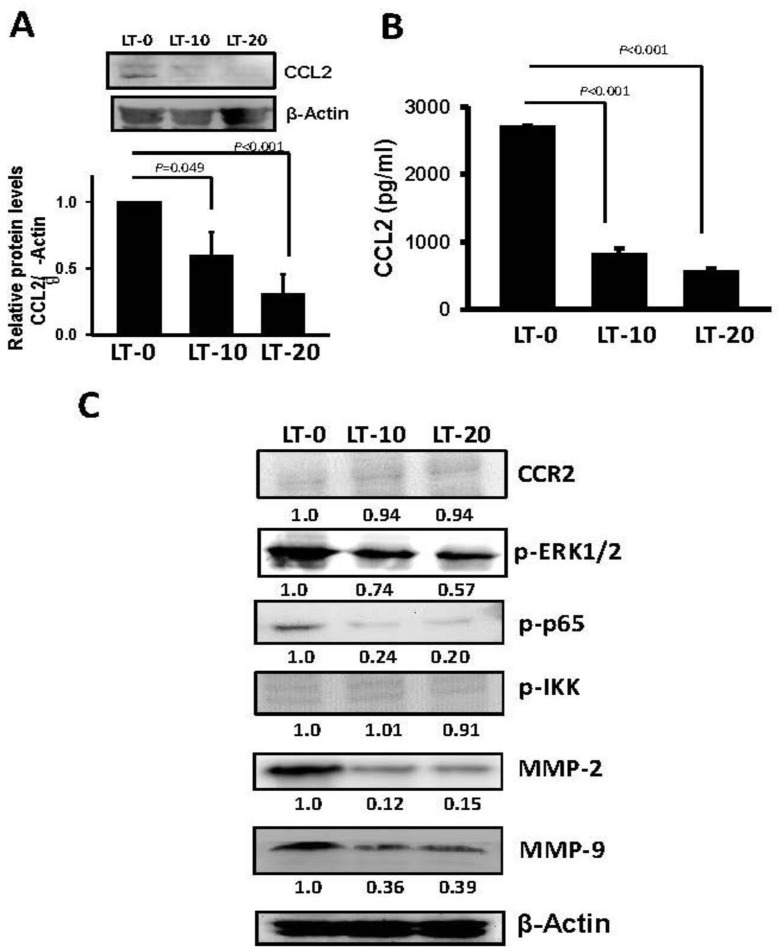
Long-term exposure to Oro-A downregulates CCL2 signaling. (**A**) The protein level of CCL2 was determined by western blotting. (**B**) The soluble form of CCL2 was examined with ELISA. (**C**) Western blotting was performed using antibodies against CCR2, phosphorylated (extracellular signal–regulated kinases 1/2) ERK1/2, phosphorylated p65, phosphorylated IKK, MMP2, and MMP9. β-Actin was used as the internal control. P values were determined using Student’s t test.

**Figure 6:**
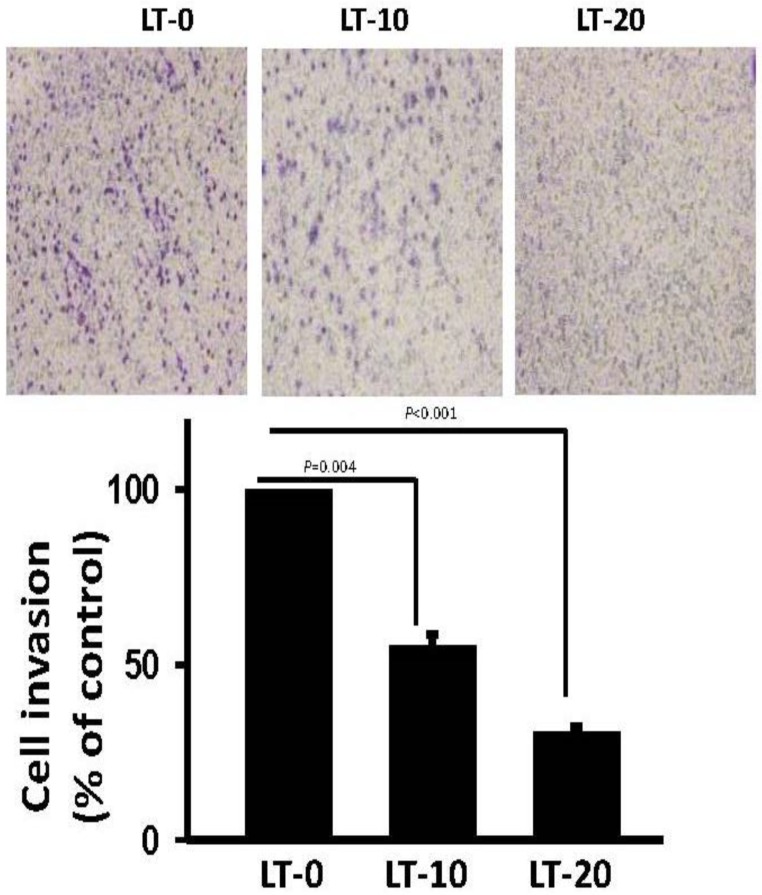
The invasiveness of LT-0, -10, and -20 cells was analyzed using Boyden chambers coated with a layer of Matrigel. All experiments were performed at least three times. P values were determined using Student’s t test.

*CCL2* has been described as regulating migration and invasion in several cancer types [29,30,31], including OSCC [32]. To evaluate the possible role of *CCL2* in long-term Oro-A-induced cell migration inhibition, we examined the expression level of *CCL2* in LT-0, -10, and -20 cells using Western blotting. As shown in Figure 5A, the protein level of CCL2 was significantly decreased in LT-10 and -20 cells compared with LT-0 cells. There was no difference in *CCR2* expression between LT-0, -10, and -20 cells, showing that long-term Oro-A exposure had no effect on *CCR2* expression. In addition, the levels of CCL2 secreted by LT-0, -10, and -20 cells were quantified with enzyme-linked immunosorbent assays (ELISAs). As shown in Figure 5B, the released soluble form of CCL2 was significantly decreased 3.2-fold and 4.7-fold in LT-10 and -20 cells, respectively, compared with LT-0 cells. A previous study showed that CCL2 regulates cell migration via the MEK/ERK1/2/NF-kB signaling pathway and MMP2/MMP9 expression [30,33]. Therefore, we determined the phosphorylation levels of ERK1/2/NF-kB and expression levels of MMP2 and MMP9 using Western blotting. As shown in Figure 5C, the phosphorylation levels of ERK1/2, and p65 were markedly inhibited in LT-10 and -20 cells compared with LT-0 cells. Similarly, the expression of MMP2 and MMP9 was markedly decreased in LT-10 and -20 cells compared with LT-0 cells. However, there was no change in the expression levels of CCR2 and phosphorylation levels of IKK in LT-10 and -20 cells compared with LT-0 cells. Subsequently, the invasion ability of LT-10 and LT-20 cells was found to be significantly lower than that of LT-0 cells (Figure 6). Taken together, long-term Oro-A exposure could suppress cell migration and invasiveness of OSCC cells. Furthermore, there was no change in the expression levels of CCL2 and CCR2 or the phosphorylation levels of ERK1/2/NF-kB signaling molecules after a 24-h exposure period (Figure S2). These results suggest that inhibitory effect of long-term Oro-A exposure on migration might be associated with *CCL2* signaling.

### 2.4. Recombinant Human *CCL2* Rescues Migration of Long-Term Oro-A Exposed OSCC Cells

**Figure 7:**
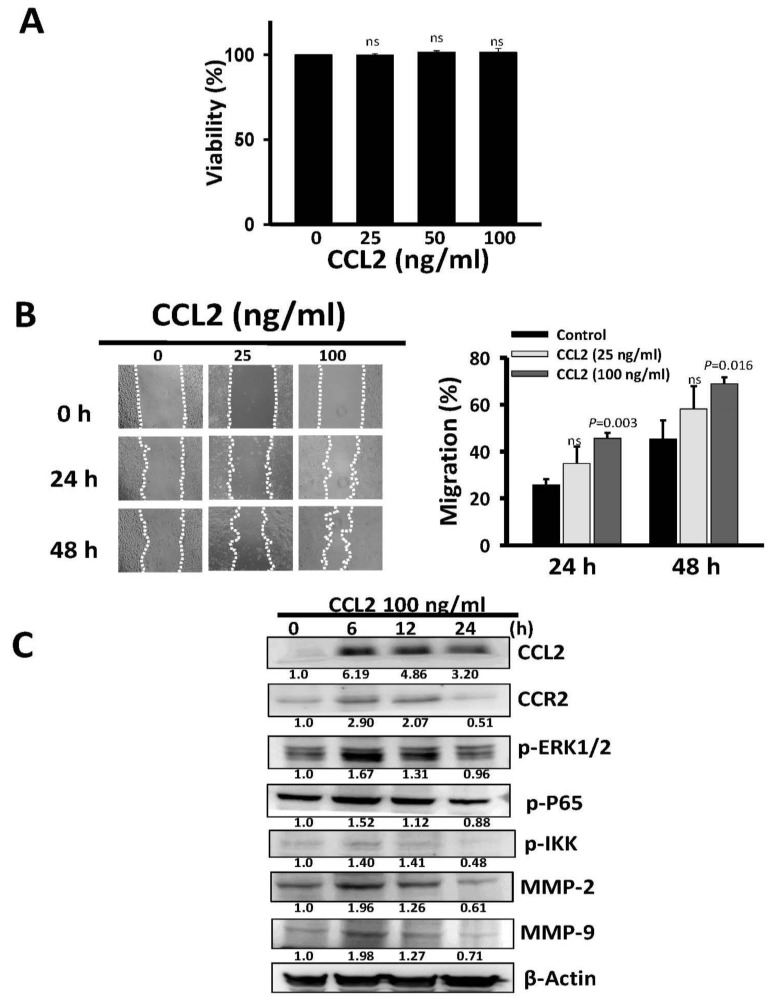
Effects of CCL2 stimulation on the proliferation and migration of LT-20 cells. (**A**) Logarithmically growing LT-20 cells were treated with CCL2 (0, 25, 50, and 100 ng/ml) for 48 h. Relative survival was assessed using the trypan blue dye exclusion method. (**B**) A monolayer of LT-20 cells was scratched with a tip and inoculated with CCL2 (0, 25 or 100 ng/ml) for 24 and 48 h. Relative migration activity was determined by wound healing assays. (**C**) Logarithmically growing LT-20 cells were treated with CCL2 (100 ng/ml) at the indicated times (0, 6, 12, and 24 h). Western blotting was performed using antibodies against CCL2, CCR2, phosphorylated ERK1/2, phosphorylated p65, phosphorylated IKK, MMP2, and MMP9. β-Actin was used as the internal control. All experiments were performed at least three times. P values were determined using Student’s t test. Ns: not significant.

To clarify whether *CCL2* has a direct effect on the ability of long-term Oro-A exposure to inhibit migration, we examined the effect of recombinant CCL2 on migration of LT-20 cells. An initial 48-h CCL2 treatment (25, 50, and 100 ng/mL) did not exert a cytotoxic effect on LT-20 cells (Figure 7A). Next, we examined the effect of CCL2 on the migration activity of LT-20 cells. The wound generated in LT-20 cells and then treated with CCL2 (25 and 100 ng/mL) for 24 and 48 h. A 24- and 48-h treatment with CCL2 (100 ng/mL) significantly enhanced the migration activity of LT-20 cells (Figure 7B). Taken together, CCL2 (100 ng/mL) did not exhibit a cytotoxic effect but could enhance migration of LT-20 cells. Meanwhile, CCL2 induced CCL2, CCR2, MMP2 and MMP9 expression and increased the phosphorylation levels of ERK1/2 and p-p65, especially at 6 h after CCL2 addition (Figure 7C). These results indicate that the CCL2/ERK1/2/NF-KB/MMP pathway plays an important role in the inhibitory effect of long-term Oro-A treatment on OSCC migration.

### 2.5. Oro-A Inhibits OSCC Metastasis In Vivo

**Figure 8:**
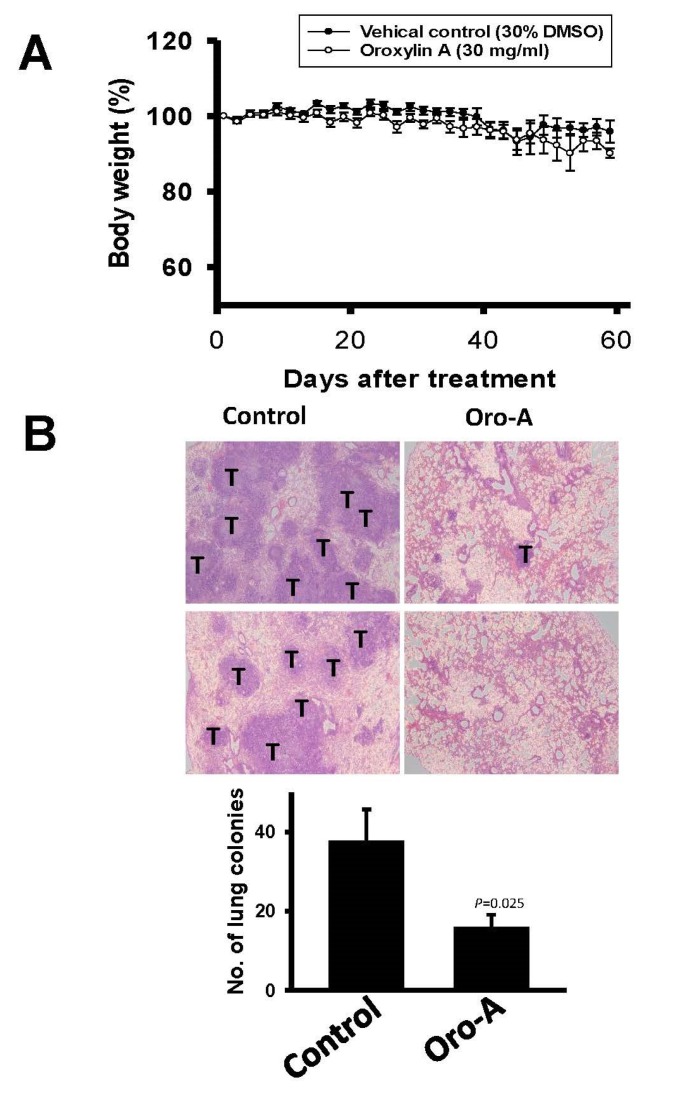
Oro-A inhibits metastasis in vivo. (**A**) Mouse body weights were measured every 2 days, and the results are presented as the means±SEM. (**B**) Representative photographs of hematoxylin- and eosin- (HE) staining lungs from 30% DMSO (vehicle control) and Oro-A group mice. “T” indicates tumor cells. Number of lung nodules in HE-stained lung sections in vehicle control and Oro-A groups. P values were determined using Student’s t test.

**Figure 9:**
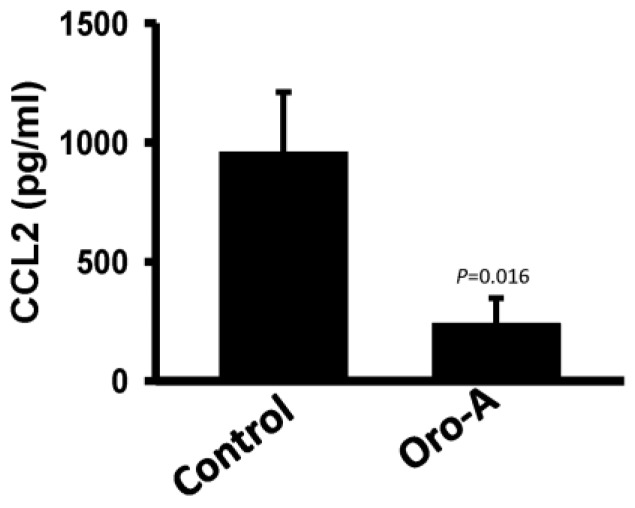
The human CCL2 levels in mice were determined with an ELISA. P values were determined using Student’s t test.

To examine the therapeutic benefit of Oro-A in OSCC metastasis, an experimental metastasis animal model was used. Mice were treated with Oro-A (30 mg/mL) or vehicle control (30% dimethyl sulfoxide, DMSO) one day prior to tail vein injection of sh-IFIT2-meta cells. Subsequently, the mice were treated with Oro-A or vehicle control at 2-day intervals. Mouse body weight was examined every other day. There was no significant difference in body weight between the Oro-A and vehicle control groups (Figure 8A). The vehicle control group had multiple tumor nodules in the lung, whereas Oro-A treatment decreased the number of tumor nodules in the lung (Figure 8B). The CCL2 levels secreted from tumors in mice were quantified with an ELISA. The CCL2 level was significantly decreased in the Oro-A group compared with the vehicle control group. The CCL2 level was significantly decreased in the Oro-A group compared with the vehicle control group (Figure 9). Moreover, Oro-A treatment ameliorated colonization outside of the lungs, particularly in the head and neck region and thoracic cavity (Table 3). These results demonstrated that Oro-A might suppress the metastatic activity of OSCC.

## 3. Discussion

This study is the first to show that long-term Oro-A exposure does not result in cytotoxic effects but can inhibit cell migration, invasion and metastasis via the CCL2 pathway in OSCC cells. CCL2/CCR2 has been implicated in the pathogenesis of several different disease processes, including vascular permeability and attraction of immune cells during metastasis [34]. This study provided evidence that Oro-A has a potential benefit against OSCC metastasis.

Oro-A is a natural product and has been reported to have multifunctional roles in anti-cancer effects [18,19,20,21,22,25,28]. However, few reports have established a long-term exposure model to address its therapeutic potential. Therefore, the present study tried to establish a long-term Oro-A exposure model to explore the detailed mechanisms by which it affects cell migration. To explore the mechanism underlying the effect of short-term Oro-A exposure on OSCC migration, we also performed a cDNA microarray analysis (data not shown). Similar to our Q-PCR results shown in Supplementary Figure S1, the expression levels of *LCN2, ID-1, MDK, S100A9*, and *CCL2* were not changed after a 24-h Oro-A exposure time, suggesting that the anti-migration mechanisms of accumulating doses of Oro-A may be different from those of short-term Oro-A exposure. The level of secreted human CCL2 was also significantly decreased in Oro-A-treated mice bearing metastatic OSCC tumors. These results suggest that a long-term exposure model may be a suitable model to demonstrate the effect of Oro-A or other natural products on cancer metastasis.

Cancer metastasis is a complex process that includes digestion of extracellular matrix, cell migration to reach the circulatory system, invasion through the vessels, and growth at secondary metastatic sites [35]. In cancer metastasis, tumor cells express chemokine receptors, and the metastatic sites secrete specific chemokines to attract the tumor cells [36]. Among the many chemokines associated with cancer progression, *CCL2*/*CCR2* signaling may be considered a major player in promoting tumorigenesis and metastasis [37,38]. CCL2 is expressed by cancer and stromal cells in the tumor microenvironment and promotes tumor cell proliferation at the primary tumor site and tumor cell migration and invasion into the surrounding extracellular matrix. During cancer metastasis, CCL2 promotes tumor cell intravasation into the circulation, likely by recruiting host myeloid cells to facilitate this process. In addition, trapping of tumor cells in small capillaries can initiate tumor cell extravasation, which is related to CCR2^+^ myeloid cells and the CCR2^+^ endothelium. CCL2 promotes tumor growth and tumor colonization at metastatic sites by recruiting additional myeloid and endothelial cells [39]. Our present study suggests that the metastasis suppression induced by long-term Oro-A treatment may be associated with *CCL2* signaling but that this treatment has no effect on CCR2 expression. Despite several uncertainties, blockade of CCL2 has been reported to be beneficial in inhibiting metastasis of various cancer types in experimental models [40,41,42]. A clinical trial has been started to assess the safety and efficacy of a CCL2 inhibitor in metastatic patients [38]. Carlumab (CNTO 888), a human monoclonal anti-CCL2 antibody, was well-tolerated but did not block the CCL2/CCR2 axis or show antitumor activity as a single agent in metastatic castration-resistant prostate cancer [43]. The benefits of Oro-A in inhibition of CCL2 and OSCC metastasis may provide new insights into the development of anti-metastatic therapies.

Matrix metallopeptidases (MMPs) are responsible for the degradation of a number of extracellular matrix (ECM) components and adhesion molecules, such as cadherins and integrins. Recent data suggest that MMPs may also function as oncogenes by promoting chromosomal instability [7]. MMP-2 and -9 are the most studied MMPs. The ECM substrates of MMP-2 are collagen types I, IV, V, VII, X, XI, and XIV and gelatin, aggrecan, elastin, fibronectin, laminin, nidogen, proteoglycan link protein, and versican [44]. The ECM substrates of MMP-9 are collagen types IV, V, VII, X, and XIV and fibronectin, laminin, nidogen, proteoglycan link protein, and versican [44]. Overexpression of MMP2 or MMP9 has been reported in various types of cancer [45,46], including OSCC [47]. MMP2 and MMP9 are known to play a role in angiogenesis, tumor growth and metastasis [48]. Development of MMP inhibitors seems to be a potential anti-metastatic strategy. Some synthetic MMP inhibitors have been tested in clinical trials and showed different levels of efficacy [49]. Oro-A can inhibit migration by suppressing the expression of MMP-2 and MMP-9 on human breast cancer cells [27]. Oro-A inhibits MMP-2/9 expression and activation by upregulating tissue inhibitor of metalloproteinase-2 (TIMP-2) and suppressing the ERK1/2 signaling pathway [28]. In addition, our results showed that long-term Oro-A treatment decreased the expression of MMP-2 and MMP-9 on OSCC cells via CCL2 signaling. Taken together, these findings strongly suggest that Oro-A has a potential anti-migration/invasion effect in vitro through suppression of MMP2 and MMP9 expression.

In addition to CCL2, we found that long-term Oro-A exposure suppressed the expression of migration-related genes, including LCN2, ID-1, MDK, and S100A9. The effect of LCN2 in cancer migration and metastasis is controversial; LCN2 overexpression in OSCC cells reduced in vitro migration and invasion and in vivo metastasis [50]. However, LCN2 is a potential therapeutic target for breast cancer metastasis [51]. ID-1 protein expression is significantly correlated with lymph node status, indicating that increased ID expression may be related to OSCC metastasis [52]. Moreover, overexpression of ID-1 is associated with tumor angiogenesis and poor clinical outcome in OSCC [53]. Enhanced expression of MDK was associated with high histological grade, late clinical stage [54] and poor survival in OSCC patients [55]. Elevated S100A9 expression in tumor stroma functions as an early recurrence marker in early-stage OSCC patients through increased tumor cell invasion, angiogenesis, macrophage recruitment and IL-6 production [56]. These results suggest that long-term Oro-A exposure not only inhibits the CCL2 pathway but also inhibits ID1, MDK, and S100A9 expression and thus has therapeutic potency against OSCC progression. Whether the involvement of *LCN2, ID-1, MDK*, and *S100A9* is associated with the anti-metastasis activity of Oro-A will be explored in future studies.

This study is the first to show that long-term Oro-A exposure does not exhibit cytotoxic effects but can inhibit cell migration via the CCL2 pathway in OSCC cells. Moreover, Oro-A inhibited in vivo metastasis in mice bearing IFIT2-depleted OSCC cells. This study provides evidence that Oro-A has potential benefits against OSCC metastasis.

## 4. Material and Methods

### 4.1. Medicine and Reagents

Oro-A was a gift from Dr. Tony Jer-Fu Lee and obtained from MedChem Express (Monmouth Junction, NJ, USA) . Extraction and purification of Oro-A was described previously [57]. Oro-A was dissolved in 100% dimethyl sulfoxide (DMSO) as a 100 mM stock solution. Recombinant Human CCL2 was obtained from PeproTech (Rocky Hill, NJ, USA).

### 4.2. Cell Culture and Treatment

The three OSCC cell lines CAL27, CA922, and SAS were used in this study. CAL27 (ATCC CRL-2095) was obtained from American Type Culture Collection (Manassas, VA, USA). SAS and CA922 were originally from the Japanese Collection of Research Bioresources (Tokyo, Japan). All OSCC cells were cultured in DMEM (Invitrogen, Carlsbad, CA, USA) containing 10% fetal bovine serum (FBS; Grand Island, NY, USA), penicillin, and streptomycin. In addition, we used sh-IFIT2-meta cells [58] derived from a metastatic tumor formed outside of the lung by a sh-IFIT2-2 clone [59], which was derived from a CAL27 subclone expressing sh-IFIT2 that was injected into the tail vein of a BALB/c nude mouse (National Laboratory Animal Center). Cells were grown in an incubator with 5% CO_2_ at 37 °C. For long-term exposure to Oro-A, 2 × 10^6^ CAL27 cells were plated onto 100-mm Petri dishes, fed daily with various concentrations of Oro-A (0, 10, or 20 μM), and subcultured at the same cell density every 3 days. Treatment was continued for 10 successive passages (30 days). These 10-passage Oro A-exposed cell populations were designated LT-0, -10, and -20 cells.

### 4.3. Measurement of Cell Viability

We determined the cytotoxicity of Oro-A using a sulforhodamine B (SRB) assay. The SRB assay was performed as described previously [60]. In brief, a minimum of four replicates of 5000 OSCC cells per well were seeded in 96-well microplates. After addition of various concentrations of Oro-A, the cells were incubated for 72 h, fixed with 10% trichloroacetic acid, and then stained with 0.4% (*w*/*v*) SRB in 10% acetic acid for 30 min. The absorbance at 510 nm was determined using a 96-well plate reader (Bio-Rad model 550; Bio-Rad, Hercules, CA). Survival curves were plotted by expressing the absorbance of treated wells as a percentage of that of control wells. The cytotoxic effect of long-term Oro-A exposure and CCL2 on OSCC cells were determined using the trypan blue dye exclusion method. Briefly, 1 × 10^5^ OSCC cells were plated in six-well plates and incubated overnight. The cells were treated with CCL2 at a 25, 50, or 100 ng/mL dilution for 48 h. Cell viability was determined by staining with trypan blue (Invitrogen).

### 4.4. Wound-Healing Assay and Transwell Invasion Assay

Wound-healing assays were performed as previously described [28]. Briefly, a monolayer of cells was ‘wounded’ using a p200 micropipette tip, and then, the cells were incubated in serum-free DMEM for 24 h in a tissue culture incubator. The wounded area was photographed at 0, 24, and 48 h and then assessed using ImageJ software (Media Cybernetics Inc., Bethesda, MD, USA). A modified Boyden chamber system was used to evaluate the effects of long-term Oro-A exposure on cell invasion. The Transwell Invasion assay was performed as described previously [59]. Briefly, 2 × 10^4^ cells were suspended in 100 mL of FBS-free medium and seeded in the upper chamber of a transwell plate onto a membrane with 8-μm pores (Corning-Costar, Corning, NY, USA) that had been precoated with Matrigel (BD-Biosciences, Palo Alto, CA, USA). The lower chamber was filled with medium containing 20% FBS. After incubation for 24 h, the cells on of the upper transwell membrane were removed using Q-tips. The cells trapped on the bottom side of the membrane were fixed and stained with crystal violet (Merck Millipore, Billerica, MA, USA) for 10 min. The number of invasive cells was determined in five random fields on each membrane, and the counts were averaged.

### 4.5. Colony Formation Assay

Colony formation assays were performed as described previously [59]. In brief, LT-0, -10, and -20 cells were seeded in 10-cm dishes at 1000 cells per well in a medium with 10% FBS. The cells were grown in the incubator for 10 days. Colonies with more than 50 cells were counted under a dissection microscope after being fixed in methanol and stained with a 10% Giemsa solution.

### 4.6. cDNA Microarray Analysis

Total RNA was isolated from LT-0, LT-10, and LT-20 cells using TRI reagent (Molecular Research Center, Cincinnati, OH, USA). The A260/A280 ratio was close to 2.0. The mRNA profiles of the LT-0, LT-10, and LT-20 cells were analyzed using Illumina HumanHT-12 v4 Expression BeadChip according to the manufacturer’s protocol (San Diego, CA, USA)) by Genetech Biotech Co., Ltd., Taiwan. Raw data were processed using Illumina Genome StudioV2011.1 software. Then, quantile normalization was applied to all of the array data to correct for bias between chips.

### 4.7. Quantitative Real-Time Polymerase Chain Reaction (Q-PCR) and Western Blotting Analysis

Q-PCR and Western blotting were performed as described previously [58]. The primers used for Q-PCR amplification are summarized in Supplementary Table S1. Primary antibodies for p-ERK1/2 and p-IKK were obtained from BD Transduction Laboratories (San Jose, California, USA). Primary antibody for ERK1/2 was obtained from Bioss (Woburn, Massachusetts, USA). Primary antibody for MMP-2 was obtained from Cell Signaling Technology (Danvers, USA). Primary antibody for MMP-9 was obtained from GenTex (Irvine, California, USA). Primary antibodies for β**-**Actin, p-p65, CCL2, CCR2 and mouse IgG (HRP), and rabbit IgG (HRP) were obtained from Abcam (Cambridge, Massachusetts, USA). Band intensities were quantified by ImageJ (NIH, Bethesda, USA) and are shown normalized to theβ-Actin lane for each target.

### 4.8. Enzyme-Linked Immunosorbent Assay (ELISA)

The conditioned medium from the LT-0, LT-10, and LT-20 cells was harvested after a 24-h incubation in medium containing 2% FBS. The sera of mice were collected from experimental metastasis model. The amount of CCL2 secreted into the conditioned medium and sera of mice was determined by a Human CCL2/MCP-1 Quantikine ELISA kit according to the manufacturer’s manual (DCP00; R&D Systems, Minneapolis, MN, USA).

### 4.9. The Experimental Metastasis Model

Animal care was approved by and followed the guidelines of the Institutional Animal Care and Utilization Committee of Tzu-Chi University. The nonobese diabetic/severe combined immunodeficiency (NOD/SCID) mice were obtained from the animal center of Tzu-Chi University (Hualien, Taiwan). The experimental metastasis model was conducted as previously described [59]. In brief, the 12- to 16-week-old NOD/SCID mice were treated with Oro-A (30 mg/mL) or vehicle control (30% DMSO) one day prior to the tail-vein injection of 5 × 10^5^ sh-IFIT2-meta cells. Subsequently, the mice were treated with Oro-A or vehicle control at 2-day intervals. The body weight of the mice was measured every other day and used as an indicator of the systemic toxicity of the treatment. After 60 days, the animals were euthanized and blood was then collected by cardiac puncture. Simultaneously, various organs from the thoracic, peritoneal and retroperitoneal cavities were removed, rinsed, fixed and subjected to pathological examination. The number of pulmonary tumor colonies was determined using a dissecting microscope. Sera were collected from the blood samples of individual mouse and maintained at –80 °C until assay.

### 4.10. Statistical Analysis

The data shown were obtained from at least three independent experiments, and all results are presented as the mean ± standard error of the mean (SEM). Differences between groups were assessed using Student’s t test analysis.

## 5. Conclusions

The present study demonstrates that long-term exposure of OSCC to non-toxic Oro-A inhibits metastasis via CCL2/ERK1/2/P65/MMP signaling. Most importantly, the present study suggests that Oro-A is a potential anti-metastasis agent for OSCC.

## Figures and Tables

**Table 1:** Top 10 downregulated migration-related candidate genes in cells with long-term Oro-A exposure.

ID	Gene	Prediction ^#^	Expression (Log 2)	Finding *
NM_005564.3	*LCN2*	Decreased	−1.613	Increased (1)
NM_181353.1	*ID1*	Decreased	−1.41	Increased (10)
NM_001012334.1	*MDK*	Decreased	−1.237	Increased (2)
NM_002965.2	*S100A9*	Decreased	−1.206	Increased (5)
NM_002982.3	*CCL2*	Decreased	−1.081	Increased (40)
NM_001038.4	*SCNN1A*	Decreased	−0.981	Increased (1)
NM_006113.4	*VAV3*	Decreased	−0.978	Increased (2)
NM_004390.2	*CTSH*	Decreased	−0.87	Increased (3)
NM_003722.3	*TP63*	Increased	−0.735	Decreased (5)
NM_004864.1	*GDF15*	Decreased	−0.729	Increased (1)

# Prediction of the effect of the target gene on OSCC migration after long-term Oro-A exposure. based on its expression direction. * The number of articles demonstrating a positive or negative relationship between the expression of the target gene and migration ability.

**Table 2:** Top 10 upregulated migration-related candidate genes in cells with long-term Oro-A exposure.

ID	Gene	Prediction ^#^	Expression (Log 2)	Finding *
NM_000758.2	*CSF2*	Increased	2.158	Increased (1)
NM_002658.2	*PLAU*	Increased	1.523	Increased (25)
NM_001945.1	*HBEGF*	Increased	1.503	Increased (1)
NM_000228.2	*LAMB3*	Increased	1.482	Increased (7)
NM_001964.2	*EGR1*	Increased	1.385	Increased (1)
NM_000584.2	*CXCL8*	Increased	1.374	Increased (10)
NM_000600.1	*IL6*	Increased	1.280	Increased (9)
NM_001005376.1	*PLAUR*	Increased	1.197	Increased (11)
NM_002203.3	*ITGA2*	Increased	1.173	Increased (1)
NM_001001392.1	*CD44*	Increased	1.139	Increased (35)

^#^ Prediction of the effect of the target gene on OSCC migration after long-term Oro-A exposure based on its expression direction. * The number of articles demonstrating a positive or negative relationship between the expression of the target gene and migration ability.

**Table 3:** Summary of the number of colonization sites outside the lung in mice injected via the tail vein with IFIT2-depleted metastatic OSCC cells and treated with vehicle control or Oro-A.

Location of Tumor	Vehicle Control (n = 6)	Oro-A (n = 6)
Head and Neck	4	1
Thoracic cavity	4	2
Subcutaneous	8	2
Skeletal muscle	8	5
Total	24	10

## Supplementary Materials

The following are available online at https://www.mdpi.com/2072-6694/11/3/353/s1: Table S1: Q-PCR primer; Figure S1: The effect of 24-h Oro-A exposure on LCN2, ID-1, MDK, S100A9, and CCL2 mRNA expression was determined by quantitative real-time polymerase chain reaction (Q-PCR); Figure S2: The effect of 24-h Oro-A exposure of on CCL2, CCR2, phosphorylated MER, IKK and P65 expression was determined by western blotting.

## Abbreviations

OSCC	oral squamous cell carcinoma
COX-2	cyclooxygenase-2
Oro-A	oroxyiln A
EGFR	epidermal growth factor receptor
SRB	sulforhodamine B
ECM	extracellular matrix
MMPs	matrix metallopeptidases
TIMP-2	tissue inhibitor of metalloproteinase-2
IPA	Ingenuity Pathway Analysis
