# Supplementary material for: Long-Term Exposure to Oroxylin A Inhibits Metastasis by Suppressing CCL2 in Oral Squamous Cell Carcinoma Cells
Source: Cancers (Basel). 2019 Mar 12;11(3):353. doi: 10.3390/cancers11030353 (PMC6468369; doi:10.3390/cancers11030353)
Supplement: Supplementary file 1 [file cancers-11-00353-s001.zip › suplementary results_/Supplementary Table 1.docx]

**Supplementary Table 1. Primer sequences for Q-PCR assay.**

| **Gene name** | **forward 5'-3'** | **reverse 5'-3'** |
| --- | --- | --- |
| LCN2 | GAAGTGTGACTACTGGATCAGGA | ACCACTCGGACGAGGTAACT |
| ID1 | CTGCTCTACGACATGAACGG | GAAGGTCCCTGATGTAGTCGAT |
| MDK | CGCGGTCGCCAAAAAGAAAG | TACTTGCAGTCGGCTCCAAAC |
| S100A9 | GGTCATAGAACACATCATGGAGG | GGCCTGGCTTATGGTGGTG |
| CCL2 | CAGCCAGATGCAATCAATGCC | TGGAATCCTGAACCCACTTCT |
| β2M | CGCTACTCTCTCTTTCTGG | ACTTCAATGTCGGATGG |
