# Supplementary figures and images for: Long-Term Exposure to Oroxylin A Inhibits Metastasis by Suppressing CCL2 in Oral Squamous Cell Carcinoma Cells
Source: Cancers (Basel). 2019 Mar 12;11(3):353. doi: 10.3390/cancers11030353 (PMC6468369; doi:10.3390/cancers11030353)

## Slide 1
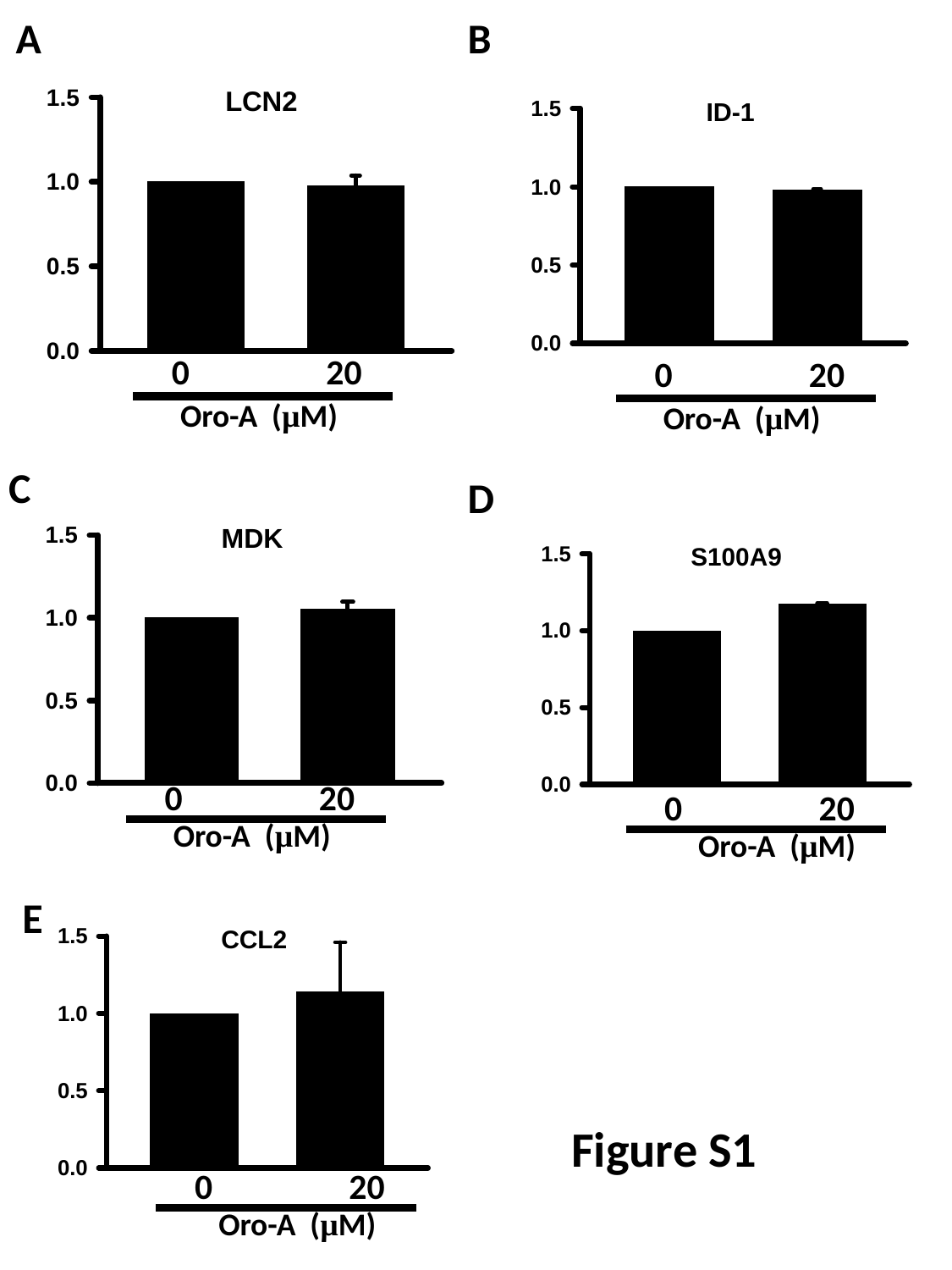

A
B
 0 20
Oro-A (µM)
 0 20
Oro-A (µM)
C
D
 0 20
 0 20
Oro-A (µM)
Oro-A (µM)
E
Figure S1
 0 20
Oro-A (µM)

Supplement: Supplementary file 1 [file cancers-11-00353-s001.zip › suplementary results_/FIGURE S1.pptx]

## Slide 1
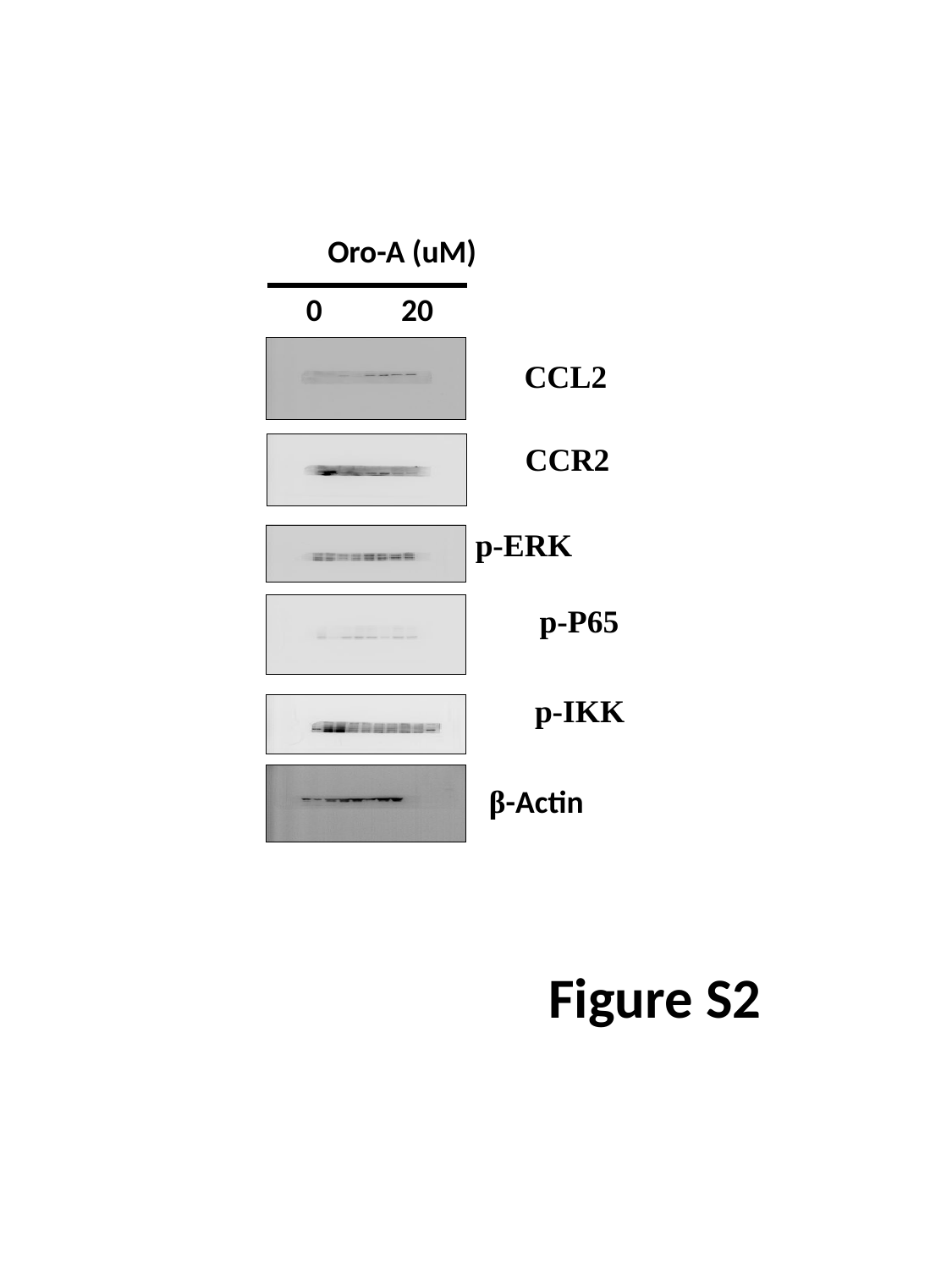

Oro-A (uM)
 0 20
CCL2
CCR2
p-ERK
p-P65
p-IKK
β-Actin
Figure S2

Supplement: Supplementary file 1 [file cancers-11-00353-s001.zip › suplementary results_/FIGURE S2.pptx]
